# Relationships of sensory processing sensitivity with creativity and empathy in an adult sample

**DOI:** 10.3389/fpsyg.2024.1465407

**Published:** 2025-01-15

**Authors:** Britta A. P. Laros-van Gorkom, Christienne G. Damatac, Inez Stevelmans, Corina U. Greven

**Affiliations:** ^1^Faculty of Psychology, Open University of the Netherlands, Heerlen, Netherlands; ^2^Department of Hematology, Radboud University Medical Center, Nijmegen, Netherlands; ^3^Department of Cognitive Neuroscience, Radboud University Medical Center, Nijmegen, Netherlands; ^4^Donders Institute for Brain, Cognition and Behaviour, Radboud University Medical Center, Nijmegen, Netherlands; ^5^Karakter Child and Adolescent Psychiatry University Centre, Nijmegen, Netherlands

**Keywords:** aesthetic sensitivity, sensory processing sensitivity, environmental sensitivity, creativity, empathy

## Abstract

**Introduction:**

Sensory Processing Sensitivity (SPS) describes individual differences in sensitivity to environments, but there is little research on potential positive correlates of SPS. Hereby we investigate whether SPS and its Aesthetic Sensitivity (AES) component are associated with different facets of creativity and empathy.

**Methods:**

Questionnaires on SPS, creativity and empathy were administered to 296 participants and data were analyzed using hierarchical multiple regression.

**Results:**

Higher SPS total and AES scores were associated with more creative ideas (SPS: β = 0.294, pfdr < 0.001; AES: β = 0.484, pfdr < 0.001). Only AES was associated with more creative activities (AES: β = 0.292, pfdr < 0.001). Furthermore, higher SPS total and AES scores were associated with more overall empathy (SPS: β = 0.428, pfdr < 0.001; AES: β = 0.373, pfdr < 0.001), affective empathy (SPS: β = 0.507, pfdr < 0.001; AES: β = 0.331, pfdr < 0.001), cognitive empathy (SPS: β = 0.2692, pfdr < 0.001; AES: β = 0.347, pfdr < 0.001), and less emotional disconnection (SPS: β = 0.234, pfdr β 0.001; AES: β = 0.210, pfdr β 0.001). Most associations remained significant after controlling for openness to experience, and the other SPS components of ease of excitation and low sensory threshold and gender, age, and education.

**Discussion:**

We conclude that SPS and AES are associated with creativity and empathy. Strengthening these positive aspects might help highly sensitive people flourish.

## Introduction

Every person is sensitive to environmental signals, although differences in the extent of sensitivity exist. Approximately 20–30% of the population score at the high end of the sensitivity continuum (Aron and Aron, 1997; Lionetti et al., 2018; Pluess, 2015; Pluess et al., 2023). Sensory processing sensitivity (SPS) is considered a personality trait that captures such inter-individual differences in sensitivity to positive and negative environmental stimuli and is ~47% heritable (Assary et al., 2021).

SPS is typically assessed with the Highly Sensitive Person Scale (Aron and Aron, 1997), which includes a total score and three subscales: ease of excitation (EOE), low sensory threshold (LST) and aesthetic sensitivity (AES) (Pluess et al., 2018). Previous research has had a strong focus on negative outcomes associated with SPS such as anxiety (Bröhl et al., 2022; Pluess et al., 2023), uncertainty (Bröhl et al., 2022), vulnerability (Pluess et al., 2023), fear and depression (Bakker and Moulding, 2012; Liss et al., 2008). In addition to correlating with the SPS total score, these negative outcomes especially correlate with two of the three subscales of the SPS total score, namely EOE (reflecting the tendency to become easily overstimulated) and LST (reflecting unpleasant sensory awareness of subtle external stimuli) (Greven et al., 2019; Smolewska et al., 2006). The questions of these subscales correspond to a negative trait cluster, which is related to, but also distinct from neuroticism (Attary and Ghazizadeh, 2021). In contrast, the AES subscale (reflecting the enjoyment of art, awareness of subtleties and deeper stimulus processing) (Aron and Aron, 1997; Aron et al., 2012) is shown to be correlated with a positive trait cluster, related to, but mostly distinct from, openness (Attary and Ghazizadeh, 2021). AES is associated with positive outcomes such as entrepreneurial intention, imagination, and enhanced intervention response (Bröhl et al., 2022; Harms et al., 2019; Pluess and Boniwell, 2015; Verheul et al., in press). AES has also been associated with adaptive coping strategies such as problem solving, cognitive restructuring, seeking social support and emotional expression and quality of life (Chacón et al., 2024). Only recently a questionnaire (SPSQ) with a more even distribution of both negative and positive items has been published (De Gucht et al., 2022). Overall, however, while multiple studies have investigated links of SPS to negative outcomes, there is research scarcity on potential positive correlates of SPS.

Personality traits can be described with the five-factor model (McCrae and Costa, 1987). Of the Big Five personality dimensions, neuroticism shows modest associations with the SPS total score and especially its LST and EOE subscales (Lionetti et al., 2019; Attary and Ghazizadeh, 2021). Openness to experience (shortened to openness) is also associated with the SPS total score (Lionetti et al., 2019), and especially its AES subscale (Lionetti et al., 2018; Pluess et al., 2018, 2023; Attary and Ghazizadeh, 2021). A recent publication supports the further differentiation of personality profiles, based on AES, LST and EOE subscales (Bürger et al., 2024), where the subgroup scoring highest on AES labeled as the Confident Sensitivity Group is more related to openness. Although it is shown that SPS predicts variance, e.g., in mental health outcomes, beyond neuroticism and openness (Greven et al., 2019; Damatac et al., 2023), it remains to be shown whether any association between SPS and positive traits are found independently of openness. This study focuses on two potential positive correlates of SPS, namely creativity and empathy, for reasons outlined as follows.

## SPS and creativity

One description of creativity is that it “*requires both originality and effectiveness*” (Runco and Jaeger, 2012, p. 92). Originality can be seen in creative ideas, whereas effectiveness can be linked to what creative ideas lead to, namely creative activities. Creativity is usually associated with artistic professions but can also be expressed as new ideas in other fields (Zaidel, 2015). A distinction can be made into Little-c creativity (everyday creativity), Big-C creativity (exceptional creativity as encountered in people such as Rembrandt), Mini-c creativity (personal and developmental creativity, e.g., learning how to play a musical instrument), and Pro-c creativity (a creative achievement not resulting in becoming famous) (Kaufman and Beghetto, 2009).

Creativity has been found to relate to increased physiological reactivity (Martindale, 1999), increased sensitivity to sensory stimuli (Carson et al., 2003), experiencing emotions more extremely and intensely (Ceci and Kumar, 2016) and being more open to one's own feelings (Kaufman, 2013). A relationship between creativity and openness to experience has been described, where creativity has been related to increased sensitivity to one's own emotions, especially among artists (Feist, 1998). What is more, interviews with highly sensitive people revealed a self-reported connection between SPS and being strongly touched by art (Smolewska et al., 2006). In other qualitative research, highly sensitive people also described creativity to be associated with their sensitivity (Bas et al., 2021). In a quantitative study such a connection was confirmed by showing that the AES subscale of the SPS total score has the highest correlation with creativity (Bridges and Schendan, 2019b) at expert and genius levels (Pro-c and Big-C creativity) (Kaufman and Beghetto, 2009). Based on this, it can be hypothesized that SPS and especially AES is related to creativity (Bridges and Schendan, 2019a; Rizzo-Sierra, 2012; Rizzo-Sierra et al., 2012). This is supported by recent findings that the subgroup of highly sensitive persons scoring highest on AES, besides having more openness in their personality profiles, also seem to be more action oriented (Bürger et al., 2024), which could possibly lead to more creative activities. It can therefore be hypothesized that SPS and AES lead to more originality, expressed in both more creative ideas, and also in more everyday creative activities.

Several questions remain unclear. Both SPS and openness to experience have been related to expert creativity (Bridges and Schendan, 2019b), however, it is unclear whether the relation between SPS and creativity is independent of openness. Furthermore, despite modest correlations between AES and the other SPS subdimensions EOE and LST, no previous study has investigated whether associations between AES and creativity are independent of the other subscales. Additionally, while SPS has been related to creativity at expert and genius levels (Bridges and Schendan, 2019b), it is unclear whether SPS also relates to creative output such as creative thinking and to performing everyday creative activities.

## SPS and empathy

Empathy is defined as “*the capacity to understand and respond to the unique affective experiences of another person”* (Decety and Jackson, 2006, p. 54). Empathy has been described as a two factor concept, each of which has distinct neural correlates: first, affective empathy, the ability to share another's emotions which may be related to mirror neurons action (Iacoboni and Dapretto, 2006) and the salience network (Stietz et al., 2019); second, cognitive empathy, the ability to understand another person's emotions and intentions, shown to involve the default mode network (Stietz et al., 2019). These two sides of empathy can be differentially expressed (Song et al., 2019). A review paper suggested that in response to social and emotional stimuli, highly sensitive persons appear to engage different brain regions involved in empathy and self-other processing (Acevedo et al., 2018), making it plausible that SPS relates to empathy-related outcomes. The more recently developed SPSQ scale has additional subscales reflecting both aesthetic and interpersonal sensitivity, making it possible to study these aspects of SPS in more detail (De Gucht et al., 2022).

For SPS, associations with more interpersonal sensitivity are demonstrated, involving both affective and cognitive empathy independent of openness to experience (Tabak et al., 2022). Semi-structured interviews with highly sensitive individuals reported a stronger emotional response to both negative and positive emotions and better understanding of the emotions of others (Bas et al., 2021). When watching different films, highly sensitive persons strongly felt the emotion that was conveyed in the film, could identify the range of emotions that they felt and also how well their own emotions matched those of the persons acting in the film (McQuarrie et al., 2023). Scoring high on SPS positive traits (including AES) but low on SPS negative traits (EOE and LST) was associated with a “lexithymic” profile (being able to feel and understand emotions) characterized by low scores on the all three subscales of the Toronto Alexithymia Scale (TAS-20) (Jakobson et al., 2024). However, highly sensitive persons experience empathy both as a deep connection with others but also as exhausting, from which they try to protect themselves (Roxburgh, 2023).

Empathy can also be divided into three components: emotional contagion or affective empathy, cognitive empathy, and emotional disconnection (Carré et al., 2013). Emotional disconnection is a process of self-protection, in which a person builds an emotional wall thereby preventing himself from being overwhelmed by the emotions of others (Batson et al., 1987; Lamm et al., 2007). It is a defense mechanism that causes a person to respond less empathetically and this feature decreases as a person matures (Bensalah et al., 2016). It is possible that highly sensitive persons have less emotional disconnection, causing them to be more easily emotionally drained. It has been shown that SPS positive traits were positively correlated with scores on the empathic concern, fantasy, and perspective taking subscales of Davis's (1983) Interpersonal Reactivity Index (with medium effect size), and SPS negative traits were positively correlated with scores on the personal distress subscale (Jakobson et al., 2024). This might lead one to predict that those who score high on both the positive and the negative SPS trait clusters would be the most likely to “feel for” others but that the high levels of personal distress they experience are emotionally draining. It has never been studied whether SPS and its AES component are associated with less emotional disconnection.

## Aims and hypotheses

In this study, we replicate and expand previous research by studying associations of SPS and its AES subscale with two different facets of potential positive characteristics, namely creativity and empathy. To this end, we use a cross-sectional sample. The first aim (1a) was to investigate the association of SPS with two key components of creativity, namely creative ideas and creative activities. Creative ideas are usually not included in the literature (Silvia et al., 2012) when measuring everyday creativity. It is, however, an important addition because deeper processing, a central characteristic of SPS, could possibly lead to creative ideas. The second aim (2a) was to evaluate the relation of SPS with empathy and its components affective and cognitive empathy and emotional disconnection. As subaims, we studied associations of the AES subscale of the SPS total score with the creativity variables (aim 1b) and empathy variables (aim 2b), correcting for openness to experience and the other SPS subscales LST and EOE, in order to study independent contributions of AES.

We hypothesized that SPS total score (H1a) and its AES subscale (H1b) would be related to more creative ideas and creative activities. We further hypothesized that the SPS total score (H2a) and its AES subdimension (H2b) would be related to more empathy, as well its affective and cognitive empathy subcomponents and less emotional disconnection. This study provides new insights into potential positive aspects of SPS, which may have practical implications for strengthening the wellbeing of highly sensitive people.

## Methods

This cross-sectional survey used an online questionnaire distributed in two ways. Firstly, via the personal network of the first author using social media and email and secondly, via flyers with a QR code to the questionnaire were distributed to individuals working in private practices providing psychological or coaching services and specializing in high sensitivity support across all regions of the Netherlands. These included various practices which focused on helping highly sensitive individuals in areas like work, study, and relationships. The flyers were intended for display in waiting areas or direct distribution to relevant clients.

A power analysis was performed beforehand using G-PowerWIN 3.1.9.4, with an effect size of 0.10, an α level of 0.05, and a desired power of 0.80, indicating that 114 participants were required for this study.

Online informed consent was obtained. This research was approved by the Research Ethical Review Committee (cETO) on May 2, 2022 nrU202203462.

### Participants

There were no inclusion or exclusion criteria for participation, except being at least 18 years old, as this was an online questionnaire and anybody could participate if they had given informed consent. Three hundred and eight nine people participated in the survey. Of these, 296 participants completed the questionnaires relating to SPS, openness and creativity, and all but 5 of these individuals also completed the empathy measure.

Out of the 296 participants, 66 (22.3%) identified as male, 224 (75.7%) as female, and for 2% this variable had missing data. The mean age was 44.3 years [standard deviation (SD) = 14.8 years; range: 18–78 years]. The distribution of participants' highest educational level was: secondary education, 16 (5.4%); vocational education, 42 (14.2%); bachelor's degree from a university of applied sciences, 75 (25.3%); master's degree from a university of applied sciences, 22 (7.4%); bachelor's degree from a university, 34 (11.5%); and master's degree from a university, 102 (34.5%).

### Questionnaires

SPS was assessed using the 12-item Highly Sensitive Person scale (12-HSP scale) (Pluess et al., 2023) in Dutch (Bröhl et al., 2022), which includes 4 items on AES, 3 on LST and 5 on EOE. An example of an AES item is: “I am deeply touched by art or music”, an example of LST is: “Intense stimuli, like hard sounds or chaotic situations I find annoying” and an example of EOE is: “I feel rushed when I have a lot to do in a short time”. The scale was scored on a 7-point Likertscale (1 = *not at all*, 7 = *extremely*) and mean scores were calculated for the SPS total score and each subscale.

Openness to experience was assessed using items from the Dutch Big Five Inventory (BFI) (Denissen et al., 2008; John et al., 1991). An example of an item is: “I see myself as someone who has a vivid imagination”. Questions were scored on a 5-point Likertscale (1 = *don't agree at all* to 5 = *completely agree*) and a mean score was calculated.

Everyday creativity was assessed with two different questionnaires: The first is the Runco Ideation Behavior Scale (RIBS) (Runco et al., 2001), measuring creative ideas and problem solving. An example of an item is: “I come up with an idea or solution that other people have never thought of”. This questionnaire had 23 items on a 5-point Likertscale (1 = *never* to 5 = *very often*). A mean score was calculated. The second questionnaire is the Biographical Inventory of Creative Behaviors (BICB) (Batey, 2007), which contained 34 items about creative activities, such as “Have you, in the past 12 months written a short story?” (0 = no, 1 = *yes)*. A BICB sumscore was calculated, to give an idea of the total amount of creative activities.

To assess empathy, the Dutch Basic Empathy Scale was used (Jolliffe and Farrington, 2006; Raadsen, 2017; Van Langen et al., 2014). This questionnaire gives rise to a total score and three subscales: affective empathy (six items; e.g., “I get easily carried away by the feelings of others”), cognitive empathy (eight items; e.g., “I understand how people feel, often before they tell me”), and emotional disconnection (six items, e.g., “My friends' feelings don't matter much to me.”) (Carré et al., 2013), scored on a 5-point Likertscale (1 = strongly disagree, 5 = strongly agree). Negatively stated items were reversed (Raadsen, 2017), so that higher scores indicated greater affective and cognitive empathy and less emotional disconnection.

### Demographic variables

Gender and age were measured as an open question. Gender was coded as two categories, as all respondents identified as either male (0) or female (1). Only six persons didn't answer the question about gender, these were therefore missing data. Education was measured as a multiple choice question. Level of education was coded as follows for the analyses: primary education (1), secondary education (2); vocational education (3); bachelor's degree from a university of applied sciences (4); master's degree from a university of applied sciences (5); bachelor's degree from a university (6); and master's degree from a university (7).

### Statistical analyses

#### Pre-processing

Analyses were performed using the Statistical Package for the Social Sciences (SPSS), version 28 for multicollinearity tests [Variance Inflation Factor (VIF)], and reliability assessment (Cronbach's alpha), and R, version 4.3.2 (2023-10-31) for Pearson correlations and network analysis. Variable distributions, outliers and assumptions for regression analysis were checked. Descriptives were reported and preliminary associations among study variables examined through Pearson correlations (*n* = 296). To visually display significant correlations between variables (*p* < 0.05) arranged into a network, we used network analysis using multidimensional scaling with the qrgaph package in R (Epskamp et al., 2018).

#### Main analyses

For all regression analyses we used the data of the questionnaire that had no missing data (*n* = 290), we used false discovery rate (FDR) to correct our *p*-values (α = 0.05), which we report as *pfdr* (Benjamini and Hochberg, 1995). To address aim 1a, regression analysis was used to examine the association between SPS as the independent variable and creative ideas and creative activities as dependent variables, resulting in two main models. Therefore, we FDR-corrected our *p*-values (α = 0.05) across two tests. We used a hierarchical approach in our regression analysis. At step one we entered SPS. At step two we entered OE and at step three we entered the demographic variables as covariates. To address aim 1b, AES as the independent variable was regressed against the same dependent variables as in aim 1a, resulting in two main models and FDR-correction applied across two tests. Also for this we used a hierarchical approach in our regression analysis. At step one we entered AES. At step two we entered OE and at step three we entered EOE, LST and the demographic variables as covariates.

To address aim 2a, regression was used to examine the association between SPS total score as the independent variable and total empathy score as the dependent variable. If the total empathy score significantly related to SPS, we then assessed the association between SPS as the independent variable and the dependent variables of affective empathy, cognitive empathy, and emotional disconnection (FDR-correction across three tests). We used a hierarchical approach in our regression analysis. At step one we entered SPS. At step two we entered OE and at step three we entered the demographic variables as covariates. To address aim 2b, AES as the independent variable was regressed against the following dependent variables: empathy, affective empathy, cognitive empathy, and emotional disconnection (FDR-correction across three tests). Also for this we used a hierarchical approach in our regression analysis. At step one we entered AES. At step two we entered OE and at step three we entered EOE, LST and the demographic variables as covariates.

## Results

### Pre-processing

Descriptive statistics are presented in Table 1. The mean total SPS score in our Dutch study sample was 4.50 (SD 1.04) [95% CI (4.38; 4.62)] and for AES 4.97 (SD 1.10) [95% CI (4.85; 5.09)] which was higher than in school- and population-based samples in the literature where a mean total SPS score of 3.99 (SD.80) [95% CI (3.91; 4.07)] and AES of 4.26 (SD 1.12) [95% CI (4.15; 4.37)] (Belgian sample) (Bröhl et al., 2022) and an AES score of 4.42 (SD 1.06) [95% CI (4.34; 4.50)] (UK sample) (Pluess et al., 2023) were found with the same questionnaire. Only the mean total SPS score of the UK sample was comparable to ours 4.34 (SD 0.89) [95% CI (4.28; 4.40)] (Pluess et al., 2023). Although cultural differences may also explain these differences, given our recruitment strategy, this likely indicates that our participants had higher than population average SPS and especially AES scores.

**Table 1:** Mean, standard deviation (SD) and Cronbach's alpha (α) of variables and covariates in our linear regression models.

**Analysis variable**		**Questionnaire**	**Mean**	**SD**	**α**
Independent	Sensory Processing Sensitivity (SPS)	12-item Highly Sensitive Person scale	4.5	1.04	0.86
	SPS-Aesthetic Sensitivity (AES)	4 items of the 12-HSP scale	4.97	1.1	0.71
Dependent	Creative ideas	Runco Ideation Behavior Scale	2.86	0.69	0.93
	Creative activities	Biographical Inventory of Creative Behaviors	7.41	4.59	0.79
	Empathy	Basic Empathy Scale	79.84	8.77	0.85
	Affective empathy	6 items of the Basic Empathy Scale	20.75	3.99	0.75
	Cognitive empathy	8 items of the Basic Empathy Scale	33.13	3.80	0.78
	Emotional disconnection	6 items of the Basic Empathy Scale	25.96	3.01	0.68
Covariates	Openness to Experience (OE)	10 items of the Big Five Inventory	3.56	0.65	0.82
	SPS-Low Sensory Threshold (LST)	3 items of the 12-HSP scale	4.31	1.47	0.67
	SPS-Ease of Excitation (EOE)	5 items of the 12-HSP scale	4.31	1.30	0.84
	Gender	Male = 0, female = 1, not stated	*n* = 66 (22.3%)	*n* = 224 (75.7%)	*n* = 6 (2%)
	Age	in years	44.3	14.8	
	Education		5.10	1.69	

The language for all questionnaires was Dutch.

**Figure 1:**
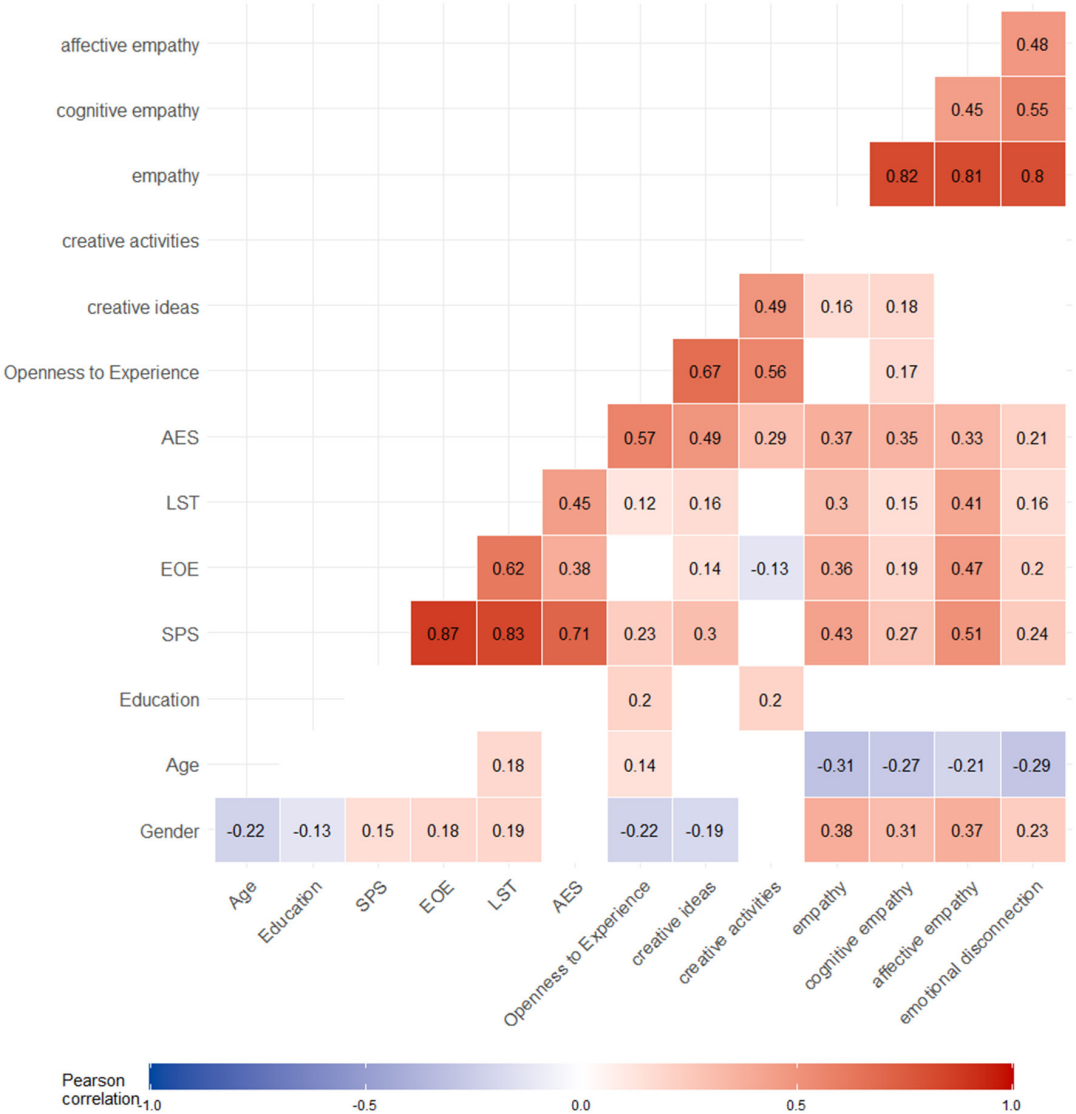
Preliminary associations through Pearson Correlations. Pearson correlations values between all analysis variables. Blank cells indicate non-significant correlations (*p* > 0.05). *N* = 296 for SPS, AES, EOE, LST, Openness to Experience, creative ideas, creative activities. *N* = 291 for empathy (total score), cognitive empathy, affective empathy and emotional disconnection. Covariate indicates variables used as covariates in subsequent regression analyses. SPS, sensory processing sensitivity; AES, aesthetic sensitivity; EOE, ease of excitation; LST, low sensory treshold.

**Figure 2:**
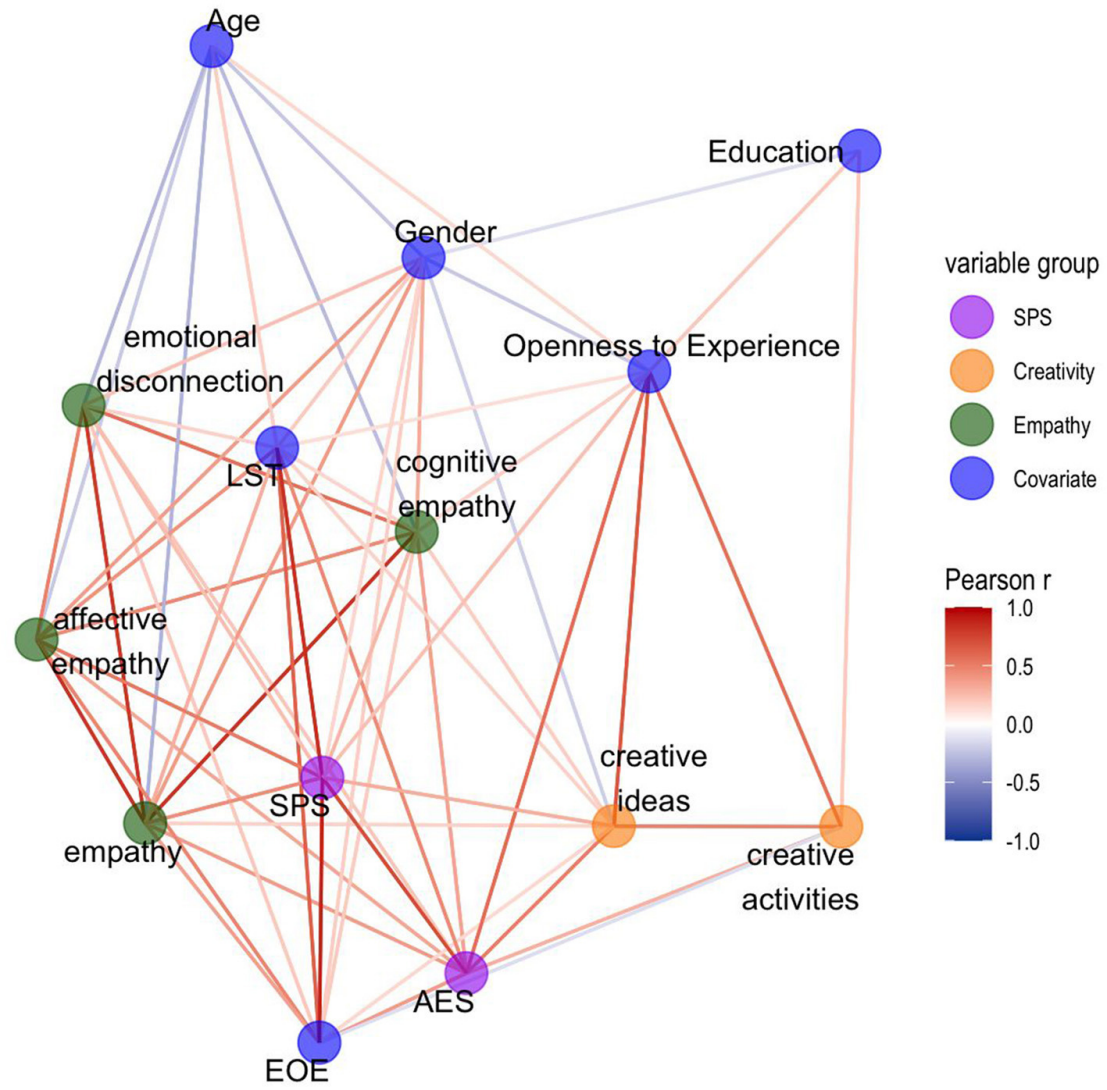
Preliminary associations through Pearson correlations and network analysis. Visual display of significant correlations between variables (*p* < 0.05) arranged into a network with minimal edges (color scale indicates Pearson correlation value). Edges (lines) represent correlations. Nodes (circles) represent questionnaires. Thicker edges reflect stronger negative (blue) or positive (red) correlations.

Pearson correlations, and visually display of significant correlations arranged into a network, revealed a positive association between higher SPS and more creative ideas, but not with creative activities (Figures 1, 2). Another notable finding was that affective empathy showed most correlations with AES, EOE and LST; whereas cognitive empathy showed small correlations with EOE and LST, but most associations with AES.

### Associations between SPS and creative ideas and creative activities (aim 1a)

Hierarchical regression analyses revealed that higher SPS associated significantly with more creative ideas, but not with more creative activities (Table 2). Running the same main association models with covariates openness in step two and openness, gender, age and education in step three revealed that especially the covariate openness reduced the strength of the association between SPS and creative ideas; however, all associations remained significant. Creative ideas showed greater associations with male gender and younger age.

**Table 2:** Associations between independent variable sensory processing sensitivity and dependent variables creative ideas and creative activities, without and with correction for covariates.

**Step**	**Variable**	**Creative ideas**				**Creative activities**			
		**B**	**Std error B**	**β**	***p***	**B**	**Std error B**	**β**	***P***
1	Constant	1.98	0.173		**< 0.001** ^*^	6.533	1.212		**< 0.001** ^*^
	SPS	0.197	0.038	0.294	**< 0.001** ^*^	0.200	0.263	0.045	0.448
2	Constant	0.013	0.190		0.945	−5.454	1.440		**< 0.001** ^*^
	SPS	0.097	0.029	0.145	**0.001** ^*^	−0.410	0.224	−0.092	0.068
	OE	0.679	0.047	0.638	**< 0.001** ^*^	4.137	0.357	0.581	**< 0.001** ^*^
3	Constant	0.581	0.223		**0.010** ^*^	−6.409	1.761		**< 0.001** ^*^
	SPS	0.119	0.029	0.178	**< 0.001** ^*^	−0.398	0.231	−0.089	0.086
	OE	0.683	0.048	0.641	**< 0.001** ^*^	4.109	0.379	0.577	**< 0.001** ^*^
	Gender	−0.201	0.073	−0.122	**0.006** ^*^	0.425	0.576	0.039	0.461
	Age	−0.010	0.002	−0.213	**< 0.001** ^*^	−0.010	0.016	−0.034	−0.665
	Education	−0.018	0.017	−0.043	0.315	0.223	0.137	0.081	0.106

*R*^2^ = 0.087 for step 1; Δ *R*^2^ = 0.384 for step 2; Δ *R*^2^ = 0.048 for step 3 (all ***ps*<** 0.001^*^) for creative ideas.

*R*^2^ = 0.002 for step 1, *p* = 0.448; Δ *R*^2^ = 0.318 for step 2, ***p*<** 0.001^*^; Δ *R*^2^ = 0.009 for step 3, *p* = 0.277 for creative activities.

pfdr, False-discovery rate (FDR) corrected *p*-values across two tests for SPS with creative ideas and creative activities. *p* < 0.05 is indicated in bold and with an asterisk^*^.

### Associations between AES and creative ideas and creative activities (aim 1b)

More AES was associated with more creative ideas and with more creative activities (Table 3). Evaluating the same main association models but separately including covariates openness, LST, EOE, gender, age and education, revealed that especially the inclusion of openness at step two led to a reduction in association strength of AES with creative ideas and creative activities; for creative ideas, however, the association remained significant, whereas for creative activities it did not, suggesting that the latter association can entirely be attributed to openness. When all covariates were added to the model in step three, AES was no longer significantly associated with neither creative ideas nor creative activities. Notably, even though AES did not account for unique variance in creative activities, EOE did (Table 3).

**Table 3:** Associations between independent variable aesthetic sensitivity and dependent variables creative ideas and creative activities, without and with correction for covariates.

**Step**	**Variable**	**Creative ideas**				**Creative activities**			
		**B**	**Std error B**	**β**	***p***	**B**	**Std error B**	**β**	***P***
1	Constant	1.381	0.162		**< 0.001** ^*^	1.447	1.183		0.222
	AES	0.305	0.032	0.484	**< 0.001** ^*^	1.230	0.237	0.292	**< 0.001** ^*^
2	Constant	0.178	0.173		0.303	−6.525	1.312		**< 0.001** ^*^
	AES	0.096	0.033	0.153	**0.004** ^*^	−0.153	0.250	−0.036	0.541
	OE	0.623	0.056	0.585	**< 0.001** ^*^	4.130	0.423	0.580	**< 0.001** ^*^
3	Constant	0.584	0.233		**0.013** ^*^	−5.465	1.828		**< 0.003** ^*^
	AES	0.062	0.037	0.099	0.097	0.206	0.293	0.049	0.484
	OE	0.662	0.059	0.621	**< 0.001** ^*^	3.713	0.462	0.521	**< 0.001** ^*^
	EOE	0.043	0.030	0.081	0.144	−0.600	0.232	−0.168	**0.010** ^*^
	LST	0.024	0.027	0.050	0.387	0.150	0.215	0.047	0.487
	Gender	−0.198	0.074	−0.120	**0.008** ^*^	0.336	0.580	0.031	0.564
	Age	−0.010	0.002	−0.212	**< 0.001** ^*^	−0.018	0.016	−0.057	0.278
	Education	−0.016	0.018	−0.038	0.383	0.223	0.140	0.082	0.111

*R*^2^ = 0.235 for step 1; Δ *R*^2^ = 0.232 for step 2; Δ *R*^2^ = 0.052 for step 3 (all ***ps*<** 0.001^*^) for creative ideas.

*R*^2^ = 0.085 for step 1, ***p* =** < 0.001^*^; Δ *R*^2^ = 0.228 for step 2, ***p*<** 0.001^*^; Δ *R*^2^ = 0.027 for step 3, ***p* =** 0.048^*^ for creative activities.

False-discovery rate (FDR) corrected *p*-values across two tests for AES with creative ideas and creative activities. *p* < 0.05 is indicated in bold and with an asterisk^*^.

### Associations between SPS and empathy (aim 2a)

Higher SPS associated significantly with more empathy, and with more affective empathy, more cognitive empathy, and less emotional disconnection (Table 4). Evaluating the same main association models with covariates openness at step two and openness, gender, age and education at step three did not change these associations. Empathy and all aspects thereof were associated with both female gender and younger age.

**Table 4:** Associations between independent variable sensory processing sensitivity and dependent variables empathy, affective empathy, cognitive empathy and emotional disconnection, without and with correction for covariates.

**Step**	**Variable**	**Empathy**				**Affective empathy**				**Cognitive empathy**				**Emotional disconnection**			
		**B**	**SE B**	**β**	***p***	**B**	**SE B**	**β**	***p***	**B**	**SE B**	**β**	***p***	**B**	**SE B**	**β**	***p***
1	Constant	63.52	2.09		**< 0.001** ^*^	11.94	0.91		**< 0.001** ^*^	28.7	0.962		**< 0.001** ^*^	22.89	0.77		**< 0.001** ^*^
	SPS	3.635	0.45	0.428	**< 0.001** ^*^	1.963	0.2	0.507	**< 0.001** ^*^	0.989	0.209	0.269	**< 0.001** ^*^	0.684	0.17	0.234	**< 0.001** ^*^
2	Constant	63.61	3		**< 0.001** ^*^	14.11	1.29		**< 0.001** ^*^	26.81	1.376		**< 0.001** ^*^	22.69	0.11		**< 0.001** ^*^
	SPS	3.64	0.47	0.428	**< 0.001** ^*^	2.074	0.2	0.536	**< 0.001** ^*^	0.893	0.214	0.243	**< 0.001** ^*^	0.674	0.17	0.231	**< 0.001** ^*^
	OE	−0.031	0.74	−0.002	0.967	−0.752	0.32	−0.12	**0.019** ^*^	0.652	0.341	0.111	0.057	0.07	0.28	0.015	0.8
3	Constant	61.73	3.23		**< 0.001** ^*^	12.88	1.46		**< 0.001** ^*^	26.16	1.545		**< 0.001** ^*^	22.69	1.27		**< 0.001** ^*^
	SPS	3.299	0.42	0.388	**< 0.001** ^*^	1.926	0.19	0.498	**< 0.001** ^*^	0.735	0.203	0.2	**< 0.001** ^*^	0.637	0.17	0.218	**< 0.001** ^*^
	OE	1.365	0.69	0.101	0.05	−0.248	0.31	−0.04	0.431	1.247	0.332	0.213	**< 0.001** ^*^	0.365	0.27	0.078	0.183
	Gender	6.002	1.06	0.287	**< 0.001** ^*^	2.368	0.48	0.249	**< 0.001** ^*^	2.44	0.505	0.27	**< 0.001** ^*^	1.194	0.42	0.166	**0.004** ^*^
	Age	−0.173	0.03	−0.291	**< 0.001** ^*^	−0.052	0.01	−0.19	**< 0.001** ^*^	−0.064	0.014	−0.25	**< 0.001** ^*^	−0.056	0.01	−0.28	**< 0.001** ^*^
	Education	0.284	0.25	0.055	0.26	0.112	0.11	0.047	0.328	0.039	0.12	0.018	0.743	0.133	0.1	0.075	0.181

*R*^2^ = 0.183 for step 1, ***p*<** 0.001^*^; Δ *R*^2^ = 0.000 for step 2, *p* = 0.967; Δ *R*^2^ = 0.194 for step 3, ***p*<** 0.001^*^ for empathy.

*R*^2^ = 0.258 for step 1, ***p*<** 0.001^*^; Δ *R*^2^ = 0.014 for step 2, ***p* =** 0.019^*^; Δ *R*^2^ = 0.113 for step 3, ***p*<** 0.001^*^ for affective empathy.

*R*^2^ = 0.072 for step 1, ***p*<** 0.001^*^; Δ *R*^2^ = 0.012 for step 2, *p* = 0.057; Δ *R*^2^ = 0.155 for step 3, ***p*<** 0.001^*^ for cognitive empathy.

*R*^2^ = 0.055 for step 1, ***p*<** 0.001^*^; Δ *R*^2^ = 0.000 for step 2, *p* = *0.800*; Δ *R*^2^ = 0.124 for step 3, ***p*<** 0.001^*^ for emotional disconnection.

pfdr: False-discovery rate (FDR) corrected *p*-values across three tests for SPS with affective empathy, cognitive empathy and emotional disconnection. *p* < 0.05 is indicated in bold and with an asterisk^*^.

### Associations between AES and empathy (aim 2b)

More AES was associated with more empathy, and with affective empathy, cognitive empathy, and less emotional disconnection (Table 5). Running the same main association models but with covariates openness at step two and openness, LST, EOE, gender, age and education, at step three, it becomes clear that both AES and EOE each account for unique variance in empathy (Table 5). All three SPS subscales account for unique variance in affective empathy (Table 5). Also the association of AES with emotional disconnection appeared reduced when including LST or EOE at step three of the analysis. However, all associations remained significant.

**Table 5:** Associations between independent variables aesthetic sensitivity and dependent variables empathy, affective empathy, cognitive empathy and emotional disconnection without and with correction for covariates.

**Step**	**Variable**	**Empathy**				**Affective empathy**				**Cognitive empathy**				**Emotional disconnection**			
		**B**	**SEB**	**β**	***p***	**B**	**SEB**	**β**	***p***	**B**	**SEB**	**β**	***p***	**B**	**SEB**	**β**	***p***
1	Constant	65.323	2.183		**< 0.001** ^*^	14.883	1.011		**< 0.001** ^*^	27.285	0.955		**< 0.001** ^*^	23.155	0.790		**< 0.001** ^*^
	AES	2.986	0.438	0.373	**< 0.001** ^*^	1.207	0.203	0.331	**< 0.001** ^*^	1.203	0.191	0.347	**< 0.001** ^*^	0.577	0.158	0.210	**< 0.001** ^*^
2	Constant	69.678	2.764		**< 0.001** ^*^	18.102	1.257		**< 0.001** ^*^	27.763	1.221		**< 0.001** ^*^	23.813	1.009		**< 0.001** ^*^
	AES	3.742	0.526	0.467	**< 0.001** ^*^	1.765	0.239	0.484	**< 0.001** ^*^	1.285	0.233	0.371	**< 0.001** ^*^	0.691	0.192	0.251	**< 0.001** ^*^
	OE	−2.256	0.891	−0.166	**0.012** ^*^	−1.668	0.405	−0.270	**< 0.001** ^*^	−0.247	0.394	−0.042	0.53	−0.341	0.325	−0.073	0.295
3	Constant	61.961	3.319		**< 0.001** ^*^	12.996	1.524		**< 0.001** ^*^	26.204	1.566		**< 0.001** ^*^	22.761	1.325		**< 0.001** ^*^
	AES	2.758	0.533	0.344	**< 0.001** ^*^	0.926	0.245	0.254	**< 0.001** ^*^	1.285	0.251	0.371	**< 0.001** ^*^	0.547	0.213	0.199	**0.011** ^*^
	OE	−0.177	0.839	−0.013	0.834	−0.518	0.386	−0.084	0.180	0.290	0.396	0.049	0.464	0.052	0.335	0.011	0.878
	EOE	0.905	0.421	0.133	**0.033** ^*^	0.695	0.194	0.225	**< 0.001** ^*^	0.049	0.199	0.017	0.806	0.161	0.168	0.069	0.340
	LST	0.392	0.390	0.065	0.315	0.434	0.179	0.159	**0.016** ^*^	−0.124	0.184	−0.048	0.501	0.082	0.156	0.040	0.599
	Gender	6.237	1.053	0.298	**< 0.001** ^*^	2.398	0.484	0.252	**< 0.001** ^*^	2.601	0.497	0.288	**< 0.001** ^*^	1.238	0.421	0.172	**< 0.004** ^*^
	Age	−0.169	0.030	−0.285	**< 0.001** ^*^	−0.052	0.014	−0.193	**< 0.001** ^*^	−0.061	−0.014	−0.238	**< 0.001** ^*^	−0.056	0.012	−0.274	**< 0.001** ^*^
	Education	0.425	0.254	0.082	0.095	0.133	0.117	0.056	0.253	0.130	0.120	0.058	2.77	0.161	0.101	0.090	0.114

*R*^2^ = 0.139 for step 1, ***p*<** 0.001^*^; Δ *R*^2^ = 0.019 for step 2, ***p* =** 0.012^*^; Δ *R*^2^ = 0.241 for step 3, ***p*<** 0.001^*^ for empathy.

*R*^2^ = 0.110 for step 1, ***p*<** 0.001^*^; Δ *R*^2^ = 0.050 for step 2, ***p*<** 0.001^*^; Δ *R*^2^ = 0.229 for step 3, ***p*<** 0.001^*^ for affective empathy.

*R*^2^ = 0.121 for step 1, ***p*<** 0.001^*^; Δ *R*^2^ = 0.001 for step 2, *p* = 0.530; Δ *R*^2^ = 0.164 for step 3, ***p*<** 0.001^*^ for cognitive empathy.

*R*^2^ = 0.044 for step 1, ***p*<** 0.001^*^; Δ *R*^2^ = 0.004 for step 2, *p* = 0.295; Δ *R*^2^ = 0.139 for step 3, ***p*<** 0.001^*^ for emotional disconnection.

pfdr: False-discovery rate (FDR) corrected p-values across three tests for AES with affective empathy, cognitive empathy and emotional disconnection. *p* < 0.05 is indicated in bold and with an asterisk^*^.

## Discussion

### General findings and implications

Where much attention has been paid to negative correlates of SPS regarding internalizing behaviors such as anxiety, stress, burnout and depression (Bröhl et al., 2022; Pluess et al., 2023), the aim of this study was to examine whether SPS and its AES subdimension were related to different facets of two potential positive correlates: everyday creativity and empathy.

With respect to creativity, our study showed that higher SPS and AES were related to more everyday creativity, confirming hypothesis 1a and 1b. This study quantitatively confirms findings from a qualitative study that found that highly sensitive people reported being creative and having many new ideas (Bas et al., 2021). Furthermore, in a study of 288 people, SPS and AES were related to creativity at expert and genius levels, both in terms of creative ideas and activities (Bridges and Schendan, 2019b). In contrast, our study focused on everyday creativity, and found AES to be associated with both creative ideas and activities, but the SPS total score only with more creative ideas. This might suggest that especially in highly sensitive people in which AES is more pronounced, creative activities are undertaken. When openness to experience was included in the model, associations of SPS and AES with creative ideas were reduced, but remained significant, whereas the small positive association of AES with creative activities became non-significant. This suggests that openness to experience partially explains associations of SPS with creative ideas, but that SPS and AES also make independent contributions. In contrast, associations between AES and creative activities were fully explained by openness to experience. In recent work, SPS was divided into a vulnerable sensitivity and a confident sensitivity group, according to personality types (Bürger et al., 2024). The vulnerable sensitivity group scored lower on AES and higher on the negative sides of SPS, EOE and LST and had in their personality domains less openness. On the other hand, the confident sensitivity group scored higher on AES and lower on EOE and LST and had more openness and openness to action in their personality profile. As we can see that less EOE is correlated with more creative activities, it could be assumed that it is especially the confident sensitivity group of SPS with less EOE and more openness that will eventually turn their creative ideas into action.

Furthermore, this study showed that SPS was positively related to empathy, confirming hypothesis 2a. Furthermore, when focusing on three aspects of empathy, we found higher SPS was related to more affective and cognitive empathy and less emotional disconnection. All models remained significant when correcting for openness. This implies that highly sensitive people more easily empathize with other people both on an emotional level, sharing the emotions of others, and on a cognitive level, understanding other people's emotions better, independent of openness. Empathy in SPS, both affective and cognitive empathy, was previously shown to be independent of openness to experience (Tabak et al., 2022). Our findings therefore confirm previous findings and extend existing literature by showing that emotional disconnection appears to be reduced in highly sensitive people. As emotional disconnection is a defense mechanism that causes a person to respond less empathetically (Bensalah et al., 2016), this suggests that highly sensitive people use fewer defense mechanisms in engaging with others, possibly leading to faster emotional exhaustion in the interaction with others (Roxburgh, 2023). It has indeed been shown that people scoring high on SPS were more easily moved emotionally and experienced more personal distress when watching tense situations in movies (McQuarrie et al., 2023), suggesting they use less emotional disconnection to protect themselves against the emotional content they experience. In addition, the personal distress highly sensitive people experience seems to be more related to LST and EOE, the negative sides of SPS (Jakobson et al., 2024). Deep processing of others' thoughts and feelings, emotional responsiveness and also the tendency to be affected by other people's emotions seems to be related to both positive and negative trait clusters of SPS (Jakobson et al., 2024)

Lastly, confirming hypothesis 2b, the AES component of SPS was positively associated with empathy, independent of openness to experience. A previous study also showed that higher AES correlated with more empathy but did not control for openness to experience (Liss et al., 2008). Our study also demonstrated that higher AES related to more affective and cognitive empathy. This was in line with a recent study showing more affective empathy for both positive and negative emotions through positive sensory responsivity (a measure that is highly correlated to AES) and cognitive empathy (Tabak et al., 2022). This link between AES and various forms of empathy could be a result of other positive features of SPS, such as social-affective sensitivity, sensory comfort or pleasure and sensory sensitivity to subtle internal and external stimuli, making it easier to pick up interpersonal feelings, relationships and atmospheres (De Gucht et al., 2022). A new finding in our study was that AES was associated with less emotional disconnection independent of openness, LST and EOE. Furthermore, affective empathy also appeared related to EOE and LST. In contrast, cognitive empathy appeared more related to AES, whereas EOE and LST showed little association with cognitive empathy. A possible conclusion is that AES is more important for cognitive empathy and, LST and EOE more for affective empathy.

### Strengths

In contrast to many previous studies that included students between 20 and 25 years, the current study included a large group of 296 adults aged 18–78 years. Results therefore may be considered relevant to various age categories and life stages. New in this study was that creative thinking was measured separately from creative activities. This is especially relevant as deeper processing is considered a central characteristic of SPS, which could lead to creative ideas. Another novel feature is that the relationship between SPS and everyday creativity instead of expert creativity was studied. Lastly this study further disentangled relations between different components of empathy and SPS, with AES being related to all aspects of empathy, whereas EOE and LST appear to play an additional role in especially affective empathy.

### Limitations

In this cross-sectional study, no statement can be made about causality, which makes it unclear whether SPS leads to more everyday creativity and empathy, or vice versa. Furthermore, the study sample as it was largely taken from the researcher's personal network, consisting of women and highly educated people. Given that advertisement of our study was partially via private practices providing psychological and coaching services for high sensitivity, the mean total SPS in our study was higher than in literature. The results of this study are therefore not generalizable but should be interpreted within the context of a sample with elevated SPS scores. Lastly, the study was based on the assumption that empathy is a positive trait; however less emotional disconnection could lead to empathic exhaustion.

## Conclusion

SPS and AES was related to everyday creativity and empathy. The association with everyday creativity was mainly seen in creative ideas, but not creative activities. Associations with empathy include both increased affective and cognitive empathy as well as decreased emotional disconnection. This last aspect could explain why empathy, often considered to be a positive quality of SPS, also comes with the risk of becoming emotionally exhausted. Future research should study the effect of strength-based interventions that stimulate everyday creativity or empathy in highly sensitive people, and examine whether this may enhance wellbeing and reduce overstimulation.

## Data Availability

The original contributions presented in the study are publicly available. This data can be found here: https://doi.org/10.34973/tah5-ec63.
